# In Silico Investigation Reveals *IL-6* as a Key Target of Asiatic Acid in Osteoporosis: Insights from Network Pharmacology, Molecular Docking, and Molecular Dynamics Simulation

**DOI:** 10.3390/medsci14010041

**Published:** 2026-01-15

**Authors:** Wanatsanan Chulrik, Aman Tedasen, Nateelak Kooltheat, Rungruedee Kimseng, Thitinat Duangchan

**Affiliations:** 1 Department of Medical Technology, School of Allied Health Sciences, Walailak University, Nakhon Si Thammarat 80160, Thailand; wanatsanan.chu@wu.ac.th (W.C.); aman.te@wu.ac.th (A.T.); nateelak.ko@wu.ac.th (N.K.); 2 Food Technology and Innovation Research Center of Excellence, Walailak University, Nakhon Si Thammarat 80160, Thailand; 3 Research Excellence Center for Innovation and Health Products (RECIHP), Walailak University, Nakhon Si Thammarat 80160, Thailand; 4 Hematology and Transfusion Science Research Center, Walailak University, Nakhon Si Thammarat 80160, Thailand; 5 Sanders Brown Center on Aging, College of Medicine, University of Kentucky, Lexington, KY 40536, USA; kim.rrd@uky.edu

**Keywords:** asiatic acid, osteoporosis, *IL-6*, network pharmacology, molecular docking, molecular dynamics simulation

## Abstract

Background/Objectives: Osteoporosis is a multifactorial skeletal disorder in which chronic inflammation, dysregulated cytokine signaling, and metabolic imbalance contribute to excessive bone resorption and impaired bone formation. Asiatic acid has demonstrated bone-protective effects, but its molecular mechanisms in osteoporosis remain incompletely understood. This study aimed to investigate the anti-osteoporotic mechanisms of asiatic acid using an integrative in silico strategy. Methods: Network pharmacology analysis was performed to identify osteoporosis-related molecular targets of asiatic acid. Molecular docking was used to predict the binding modes and affinities between asiatic acid and its target proteins. Molecular dynamics simulation was used to assess the structural stability and interaction persistence of the asiatic acid–protein complex. Results: Network pharmacology identified 135 overlapping targets between asiatic acid and osteoporosis, with *IL-6*, *STAT3*, *PPARG*, and *NFKB1* emerging as key hubs. KEGG analysis indicated the PPAR signaling pathway as a potential mechanism underlying the anti-osteoporotic effect. Molecular docking showed strong binding energies of asiatic acid with all predicted target proteins, with the highest affinity observed for *IL-6*, involving key residues ASN61, LEU62, GLU172, LYS66, and ARG168. Consistently, molecular dynamics simulation confirmed stable binding of asiatic acid to *IL-6*, with persistent interactions with ASN61, LYS66, LEU62, LEU64, and GLN154 mediated by hydrogen bonds, water bridges, and hydrophobic interactions. Conclusions: This integrative in silico study provides mechanistic insight into the potential anti-osteoporotic actions of asiatic acid, implicating *IL-6* as a plausible upstream molecular target. These results establish a robust mechanistic framework for future translational studies exploring asiatic acid as a natural therapeutic candidate for osteoporosis.

## 1. Introduction

Osteoporosis is a prevalent metabolic bone disorder characterized by decreased bone mass and deterioration of bone microarchitecture, leading to increased fracture risk and substantial morbidity and mortality worldwide. With population aging, osteoporosis has emerged as a major public health concern, particularly among postmenopausal women and older adults [1,2]. Although estrogen deficiency is a primary driver of postmenopausal osteoporosis, accumulating evidence indicates that osteoporosis is a multifactorial disease involving chronic low-grade inflammation, dysregulated cytokine signaling, and altered metabolic regulation of bone remodeling [3,4,5,6]. Inflammatory cytokines play a pivotal role in bone homeostasis by modulating the balance between osteoblast-mediated bone formation and osteoclast-driven bone resorption. Among these, interleukin-6 (*IL-6*) is a key mediator linking inflammation to bone loss, promoting osteoclastogenesis through activation of the gp130/Janus kinase (JAK)/signal transducer and activator of transcription 3 (*STAT3*) pathway and upregulation of receptor activator of nuclear factor-κB ligand (RANKL) [7,8]. In parallel, nuclear factor-κB (*NF-κB*) signaling is essential for osteoclast differentiation downstream of RANKL–RANK interaction and is further amplified under inflammatory and estrogen-deficient conditions [9]. Metabolic regulators such as peroxisome proliferator-activated receptor γ (*PPARγ*) also contribute to osteoporosis by shifting mesenchymal stem cell differentiation toward adipogenesis at the expense of osteoblastogenesis, thereby impairing bone formation [10]. These pathways are highly interconnected, underscoring the complex, multi-target nature of osteoporosis pathophysiology.

Current pharmacological treatments for osteoporosis, including bisphosphonates, selective estrogen receptor modulators, and monoclonal antibodies, effectively reduce fracture risk but are associated with limitations such as adverse effects, high cost, and reduced long-term adherence [11,12]. Consequently, there is growing interest in identifying safer therapeutic agents capable of modulating multiple disease-relevant pathways simultaneously. Natural compounds with pleiotropic biological activities have attracted particular attention as potential adjuncts or alternatives for osteoporosis management [13]. Asiatic acid, a pentacyclic triterpenoid derived from *Centella asiatica*, has been reported to exert anti-inflammatory, antioxidant, and metabolic regulatory effects in various disease models [14,15,16]. Emerging experimental evidence suggests that asiatic acid can attenuate osteoclastogenesis and protect against bone loss, primarily through suppression of *NF-κB* signaling and modulation of adipogenic differentiation [17,18]. However, these studies have largely focused on individual pathways or downstream effects, and a comprehensive understanding of asiatic acid’s molecular targets and system-level mechanisms in osteoporosis remains lacking. In particular, whether asiatic acid directly interacts with upstream inflammatory mediators such as *IL-6* and *STAT3*, and how these interactions integrate with known *NF-κB* and PPAR signaling networks, has not been systematically explored.

Network pharmacology, combined with molecular docking and molecular dynamics simulation, offers a powerful systems-level approach to elucidate the multi-target and multi-pathway mechanisms of bioactive compounds in complex diseases [19,20,21]. By integrating target prediction, pathway enrichment, structural interaction analysis, and dynamic stability assessment, this framework enables mechanistic hypothesis generation beyond single-target paradigms.

Accordingly, the aim of the present study was to elucidate the molecular mechanisms underlying the anti-osteoporotic potential of asiatic acid using an integrative in silico strategy. Specifically, network pharmacology, molecular docking, and molecular dynamics simulation were employed to predict the putative targets of asiatic acid, evaluate its binding affinities with osteoporosis-related proteins, and assess the stability of the asiatic acid–protein complex. Through these integrative computational approaches, this study seeks to provide mechanistic insight into the potential of asiatic acid as a natural therapeutic candidate for the prevention and management of osteoporosis.

## 2. Materials and Methods

### 2.1. Network Pharmacology Analysis

#### 2.1.1. Prediction of Asiatic Acid and Osteoporosis Targets

Network pharmacology analysis was conducted to identify potential molecular targets associated with asiatic acid and osteoporosis. The identification of asiatic acid targets (species: *Homo sapiens*) was carried out using three databases, namely SwissTargetPrediction (https://www.swisstargetprediction.ch; accessed on 5 March 2025), the Similarity ensemble approach (SEA) (https://sea.bkslab.org; accessed on 5 March 2025), and SuperPred (https://bio.tools/superpred; accessed on 5 March 2025). Osteoporosis-related targets were retrieved from the GeneCards database (https://www.genecards.org; accessed on 5 March 2025). The overlapping targets between asiatic acid and osteoporosis were visualized using a Venn diagram online tool (https://bioinformatics.psb.ugent.be/webtools/Venn/; accessed on 5 March 2025).

#### 2.1.2. Gene Ontology (GO) and Kyoto Encyclopedia of Genes and Genomes (KEGG) Pathway Enrichment Analysis

GO and KEGG pathway enrichment analyses were performed using ShinyGO version 0.82 (http://bioinformatics.sdstate.edu/go/; accessed on 5 March 2025). Enrichment results were evaluated based on fold enrichment values and −log10 (false discovery rate, FDR) values, with *p* < 0.05 considered statistically significant. Cytoscape version 3.10.3 (www.cytoscape.org; accessed on 5 March 2025) was used to visualize the networks between asiatic acid and its anti-osteoporotic targets. The cytoHubba plugin (https://apps.cytoscape.org/apps/cytohubba; accessed on 5 March 2025) was employed to identify hub targets based on degree centrality.

#### 2.1.3. Construction of Protein–Protein Interaction (PPI) Network

PPI network plays a pivotal role in a variety of cellular biological processes, which are crucial for understanding molecular mechanisms, cellular processes, and pathways, thereby facilitating drug discovery [22]. We therefore constructed a PPI network using the STRING database (https://string-db.org; accessed on 5 March 2025) with the species set to *Homo sapiens* and a medium-confidence score (>0.4) to balance interaction reliability and network completeness in the context of osteoporosis. The resulting network enabled the identification of highly connected proteins, which were considered hub targets based on their degree values.

### 2.2. Molecular Docking

#### 2.2.1. Protein Preparation

Molecular docking analysis was performed using AutoDockTools (ADT) version 4.2 [23]. Three-dimensional (3D) crystal structures of 10 target proteins (receptors) were obtained from the Research Collaboratory for Structural Bioinformatics (RCSB) Protein Data Bank (PDB) (https://www.rcsb.org, accessed on 5 March 2025) with a resolution of less than 3.5 Å, in PDB format. Protein structures were prepared by removing ligands/inhibitors and water molecules using the Visual Molecular Dynamics (VMD) software version 2.0.0. Polar hydrogen atoms were subsequently added to the proteins using ADT software version 4.2. The prepared protein structures were saved in PDBQT format files (PDB, Partial Charge (Q), and Atom Type (T)).

#### 2.2.2. Ligand Preparation

The 3D structures of asiatic acid and native ligands were retrieved from the PubChem database (https://pubchem.ncbi.nlm.nih.gov, accessed on 5 March 2025). Ligand structures were converted to PDB format using the online SMILES Translator and Structure File Generator (https://cactus.nci.nih.gov/translate/, accessed on 5 March 2025). Polar hydrogen atoms were added to the ligands and further prepared in PDBQT format files using ADT.

#### 2.2.3. Docking Protocol

Semi-flexible molecular docking was conducted, in which the receptor proteins were treated as rigid while the ligand molecules remained flexible. Grid maps were generated using ADT. Partial atomic charges of the proteins and ligands were assigned using the Gasteiger–Marsili method [24]. A blind docking strategy was employed; therefore, a cubic grid box was defined to encompass the entire receptor structure for each target protein, without assuming a predefined active pocket. The grid center was positioned at the geometric center of each protein, and the grid dimensions were individually adjusted to fully cover the receptor surface, allowing exploration of all potential ligand-binding regions. Docking simulations were performed using the Lamarckian genetic algorithm with 50 independent runs and a population size of 200 to identify energetically favorable binding sites for asiatic acid. The docked conformation with the lowest binding energy (ΔG_docking_; kcal/mol) and the corresponding inhibitory constant (K_i_) in the most populated cluster was selected for each protein. Docked conformations with the lowest binding energies and interaction residues were visualized using BIOVIA Discovery Studio Visualizer (Dassault Systèmes BIOVIA Corp., San Diego, CA, USA).

### 2.3. Molecular Dynamics Simulation

**Figure 1:**
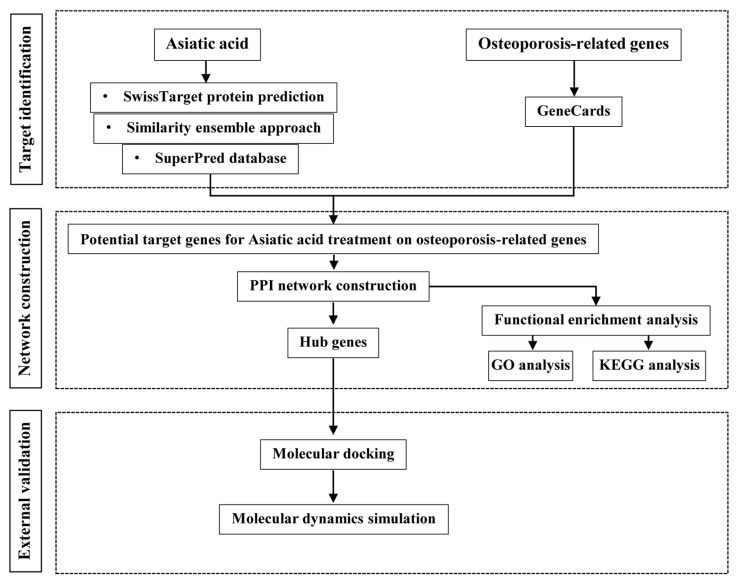
The general workflow illustrating network pharmacology, molecular docking, and molecular dynamics simulation studies to investigate the effects of asiatic acid on osteoporosis.

Molecular dynamics (MD) simulation, a powerful computational technique, simulates the physical movements of atoms and molecules over time and provides insights into the dynamic behavior and properties of biomolecular systems under various conditions [25]. MD simulation was conducted to further investigate the stability, interaction patterns, and binding dynamics of asiatic acid and MD2-*TLR4*-IN-1 (a native *IL-6* inhibitor) with the *IL-6* protein. Discovery Studio’s protein preparation technique was used to prepare the *IL-6* protein structure, including the protonation state of any ionizable side chain. The AMBER ff14SB and generalized AMBER force field were used for protein and ligand, respectively. For the simulations, the system was built using the TIP3P solvation model and centered within an orthorhombic cubic box measuring 10 Å × 10 Å × 10 Å, filled with single-point charge water molecules. The system was neutralized by adding random counterions (sodium and calcium ions), and an isosmotic state was maintained by adding 0.15 M NaCl. The molecular dynamics simulation was performed under an NPT (constant number of particles, constant pressure, and constant temperature) ensemble at 310 K and 1.01 bar, with a 200-nanosecond simulation duration. Finally, root mean square deviation (RMSD) and root mean square fluctuation (RMSF) were calculated to gain insight into the stability and identify flexible regions in the *IL-6*–asiatic acid and *IL-6*–MD2-*TLR4*-IN-1 complexes throughout the simulation. A detailed flowchart depicting the study process is presented in Figure 1.

## 3. Results

### 3.1. Network Pharmacology Analysis of Asiatic Acid Targets Related to Osteoporosis

**Figure 2:**
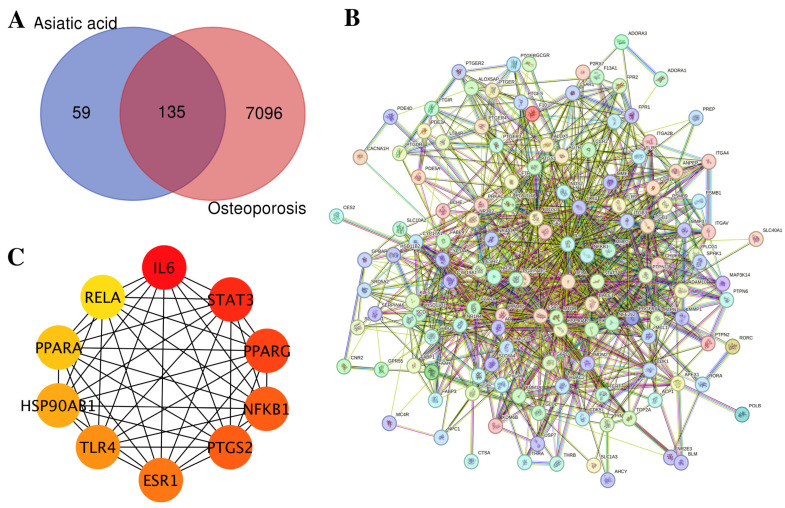
Network pharmacology analysis of asiatic acid in relation to osteoporosis. (**A**) Venn diagram illustrating the overlapping protein targets between asiatic acid and osteoporosis-related genes. (**B**) PPI network comprising 135 shared targets, generated using the STRING database. (**C**) The top 10 hub genes identified using the cytoHubba plugin in Cytoscape based on degree scores; node color reflects hub gene ranking (red = highest, yellow = lowest).

A network pharmacology analysis was conducted to investigate the potential molecular mechanisms underlying the anti-osteoporotic effects of asiatic acid. A total of 194 predicted gene targets of asiatic acid were identified using SwissTargetPrediction, SEA, and SuperPred. In parallel, 7231 osteoporosis-related targets were retrieved from the GeneCards database. Intersection analysis revealed 135 overlapping targets, suggesting that asiatic acid may exert multi-target therapeutic effects (Figure 2A). The top 10 of 135 overlapping targets is presented in Table 1, whereas the complete list of all 135 overlapping asiatic acid–osteoporosis targets is provided in Table S1. The STRING database revealed a PPI network comprising 135 nodes and 1019 edges, with an average node degree of 15.1 (Figure 2B). Subsequent degree centrality analysis using the cytoHubba plugin in Cytoscape identified the top 10 hub genes from the PPI network based on their topological importance. The hub genes were ranked in descending order according to their cytoHubba scores as follows: *IL-6*, *STAT3*, *PPARG*, *NFKB1*, *PTGS2*, *ESR1*, *TLR4*, *HSP90AB1*, *PPARA*, and *RELA*, indicating their relative centrality and potential regulatory significance within the network (Figure 2C). In the hub gene network, node color reflects cytoHubba ranking (red = highest, yellow = lowest) (Figure 2C).

**Figure 3:**
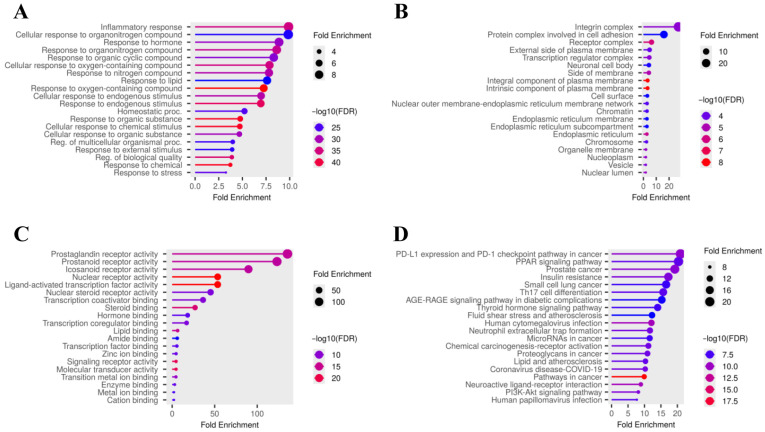
The top 20 GO enrichment terms and potential KEGG pathway enrichment of screened asiatic acid target genes in osteoporosis-related genes. GO analysis of (**A**) biological processes, (**B**) cellular components, and (**C**) molecular functions. (**D**) KEGG pathway enrichment analysis showing osteoporosis-related pathways potentially modulated by asiatic acid. Fold enrichment and −log10(FDR) values are shown to indicate statistical significance.

To explore the biological functions of asiatic acid in relation to osteoporosis-related targets, a comprehensive GO enrichment analysis was performed on the 135 candidate targets using ShinyGO. The analysis yielded 1789 significant GO terms in total, encompassing 1000 biological process terms, 228 cellular component terms, and 561 molecular function terms. The top 20 GO biological process enrichment terms were primarily associated with inflammatory response, response to hormone, response to oxygen-containing compound, response to organic cyclic compound, and homeostatic process (Figure 3A and Table 2). Cellular component analysis revealed enrichment in the integrin complex, receptor complex, protein complex involved in cell adhesion, and external side of the plasma membrane (Figure 3B and Table 2). Molecular function analysis identified nuclear steroid receptor activity, nuclear receptor activity, ligand-activated transcription factor activity, and hormone binding (Figure 3C and Table 2). Moreover, KEGG pathway analysis was performed to elucidate the relevance of asiatic acid to osteoporosis-related pathways. Among the 184 identified pathways, the top 20 pathways are presented in Figure 3D and Table 2, including the PI3K–Akt signaling pathway, PPAR signaling pathway, Th17 cell differentiation, and the thyroid hormone signaling pathway. These findings highlight the pleiotropic potential of asiatic acid in regulating several osteoporosis-associated pathways through multiple targets.

### 3.2. Molecular Docking Analysis of Asiatic Acid with Osteoporosis-Related Proteins

**Figure 4:**
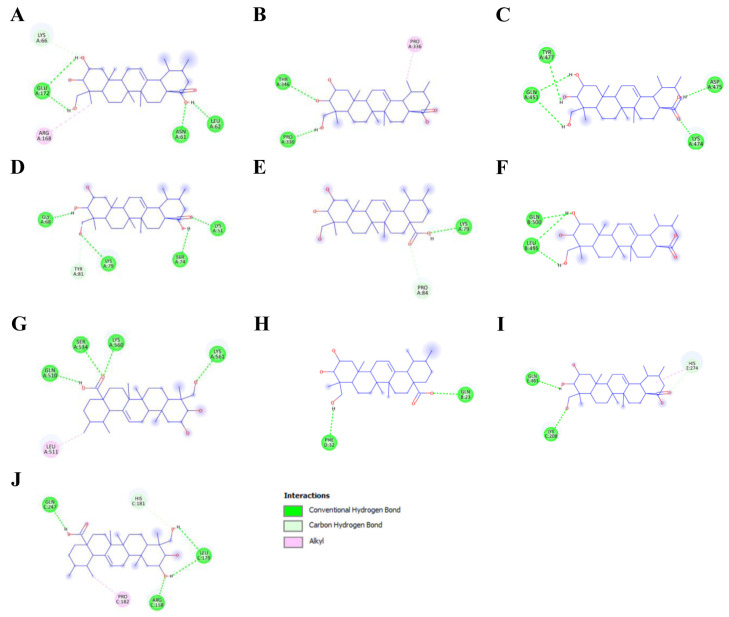
2D molecular docking analysis illustrating the interactions between asiatic acid (blue) and its osteoporosis protein targets. Panels illustrate interaction maps with (**A**) *IL-6*, (**B**) *STAT3*, (**C**) *PPARγ*, (**D**) *NF-κB* p105, (**E**) COX-2, (**F**) ESRα, (**G**) *TLR4*, (**H**) HSP90-β, (**I**) PPARα, and (**J**) *NF-κB* p65.

**Figure 5:**
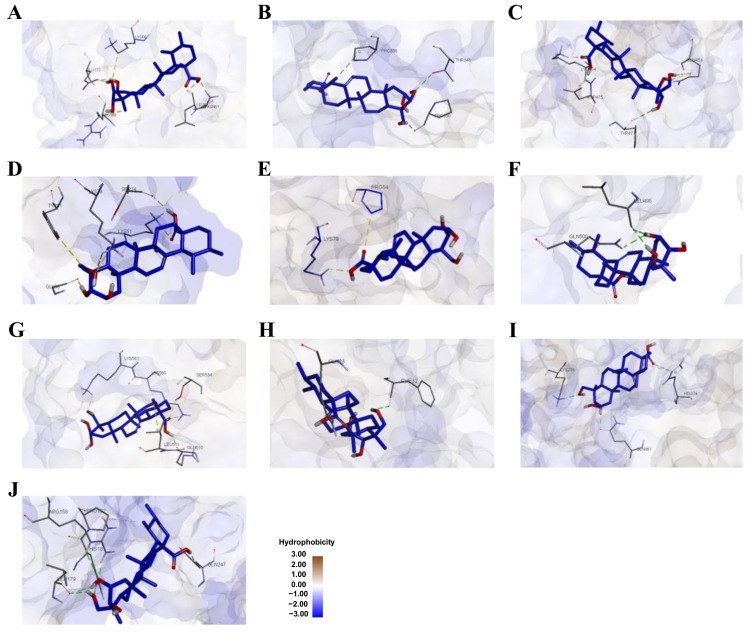
3D molecular docking analysis illustrating the interactions between asiatic acid (blue) and its osteoporosis protein targets. Panels illustrate interaction maps with (**A**) *IL-6*, (**B**) *STAT3*, (**C**) *PPARγ*, (**D**) *NF-κB* p105, (**E**) COX-2, (**F**) ESRα, (**G**) *TLR4*, (**H**) HSP90-β, (**I**) PPARα, and (**J**) *NF-κB* p65. Pink, green, and purple dashed lines represent hydrophobic interactions, hydrogen bonds, and π–σ interactions, respectively.

Based on the network pharmacology analysis, 10 key osteoporosis-related proteins encoded by predicted target genes, including *IL-6*, *STAT3*, *PPARG*, *NFKB1*, *PTGS2*, *ESR1*, *TLR4*, *HSP90AB1*, *PPARA*, and *RELA*, were selected for molecular docking analysis. Molecular docking was performed using ADT software to predict the binding interactions between asiatic acid and the selected targets. The results showed that asiatic acid exhibited favorable binding affinities toward all selected proteins, with the binding energies in the most populated cluster lower than −5.00 kcal/mol, indicating strong binding activity. Among these targets, asiatic acid showed the strongest binding affinity toward *IL-6* (−8.64 kcal/mol), followed by *PPARγ* (−7.92 kcal/mol), *NF-κB* p105 (−7.70 kcal/mol), cyclooxygenase-2 (COX-2) (−6.86 kcal/mol), PPARα (−6.41 kcal/mol), *STAT3* (−6.17 kcal/mol), *NF-κB* p65 (−6.15 kcal/mol), estrogen receptor α (ESRα) (−5.98 kcal/mol), toll-like receptor 4 (*TLR4*) (−5.87 kcal/mol), and heat shock protein 90 β (HSP90-β) (−5.57 kcal/mol). Notably, asiatic acid exhibited comparable or lower binding energies than the corresponding native ligands for several key targets, particularly *STAT3*, *PPARγ*, *NF-κB* p105, ESRα, *TLR4*, and *NF-κB* p65. Detailed binding poses and key amino acid interactions of asiatic acid with the osteoporosis-related proteins are illustrated in Figure 4 and Figure 5, with quantitative docking parameters summarized in Table 3.

For *IL-6*, asiatic acid occupied a binding pocket similar to that of the native ligand and interacted with several conserved residues, including GLU172, LYS66, and ARG168 (Table 4 and Table S1). Specifically, asiatic acid formed hydrogen bonds with ASN61, GLU172, LEU62, and LYS66, along with a hydrophobic interaction involving ARG168. In addition, asiatic acid formed stable interactions with *STAT3*, including hydrogen bonds with THR346 and PRO330, along with hydrophobic interactions involving PRO336 (Figure 4 and Figure 5 and Table 4). Native ligand interaction details are provided in Figures S1 and S2 and Table S1. Collectively, these findings support the role of asiatic acid in targeting key proteins associated with osteoporosis, particularly within the *STAT3*, *NF-κB*, and PPAR signaling pathways.

### 3.3. Molecular Dynamics Simulations of Asiatic Acid and IL-6

**Figure 6:**
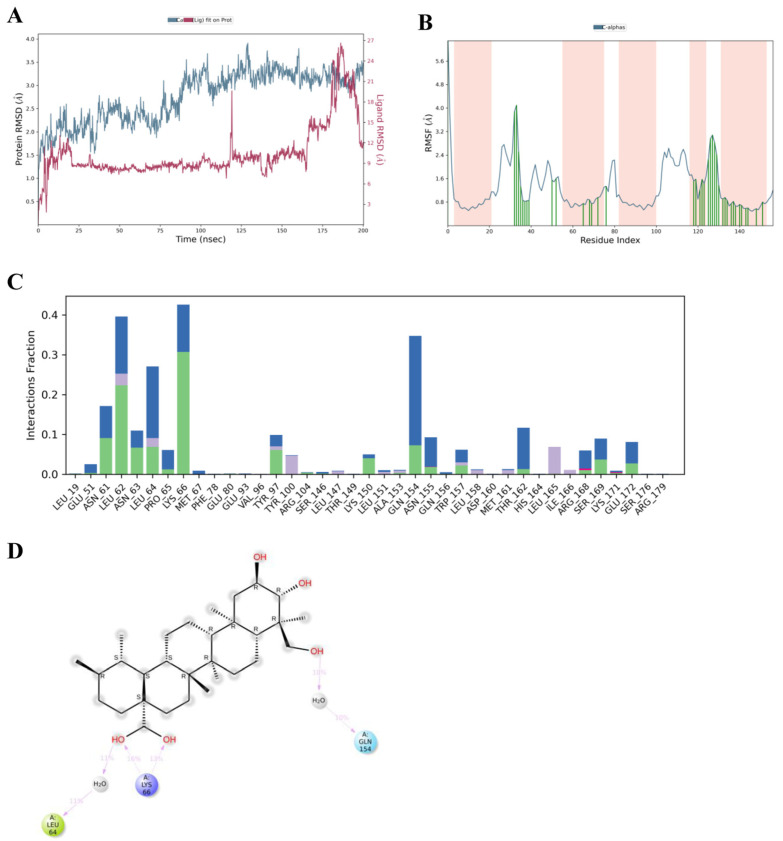
Molecular dynamics simulation of asiatic acid-*IL-6* complex. (**A**) RMSD plot showing structural stability of *IL-6* (blue) and asiatic acid (red) over 200 ns simulation. (**B**) RMSF plot indicating fluctuation of Cα atoms across residues; pink shaded areas indicate binding site regions. (**C**) Interaction fraction plot showing frequency of ligand–residue contacts throughout simulation as Hydrogen Bonds (Green), Hydrophobic (Purple), Ionic (Pink), and Water Bridges (Blue). (**D**) Final 2D binding pose of asiatic acid with *IL-6* showing polar interaction with GLN154 (Blue), electrostatic interaction with the positively charged residue LYS66 (purple), hydrophobic interactions with LEU64 (green), and water molecules involved in water-bridge interactions (gray).

The molecular docking analysis demonstrated a strong binding interaction between asiatic acid and *IL-6*. To further evaluate the stability of this interaction and validate the docking predictions under dynamic conditions, molecular dynamics simulations were performed. The structural stability and dynamic behavior of the asiatic acid-*IL-6* complex were assessed using RMSD, RMSF, and ligand–residue interaction analyses. During the simulation, the RMSD of the *IL-6* backbone increased rapidly during the initial equilibration phase (0–20 ns) and subsequently stabilized, fluctuating between 2.5 and 3.5 Å throughout the trajectory. This behavior indicates moderate conformational flexibility while maintaining overall structural stability. The RMSD of asiatic acid (Lig fit Prot) in complex with *IL-6* remained relatively stable during most of the simulation, with average values of approximately 2–4 Å, suggesting that asiatic acid maintained a stable binding conformation within the *IL-6* binding site. Transient RMSD increases were observed at approximately 120 ns and again during the late simulation phase (170–190 ns), suggesting short-lived conformational rearrangements of asiatic acid within the binding pocket rather than complete dissociation (Figure 6A). Overall, the RMSD profiles indicate a sustained association of asiatic acid with *IL-6* over the 200 ns trajectory.

RMSF analysis revealed that most *IL-6* residues exhibited fluctuations below 1.5 Å, indicating a generally rigid protein structure that may support stable ligand binding (Figure 6B). However, increased flexibility was observed in specific loop regions, particularly around residues 35–45 and 120–130. Ligand–residue interaction fraction analysis demonstrated that asiatic acid formed persistent contacts with several key *IL-6* residues throughout the simulation. Notably, residues GLN154, ASN61, LEU62, LEU64, PRO65, and LYS66 exhibited the highest interaction fractions, indicating their major contribution to ligand stabilization within the binding pocket. Additional interactions were observed with ASN63, ASN155, TRP157, THR162, SER169, LEU165, GLU51, GLU172, LYS150, TYR97, TYR100, PRO65, and ARG168, suggesting an extended interaction network involving hydrophobic contacts, hydrogen bonds, and water bridges (Figure 6C). A 2D interaction map from the molecular dynamics simulation further confirmed that asiatic acid formed stable hydrogen bonds with LYS66, highlighting its anchoring role within the *IL-6* AB-loop region. Moreover, water-mediated contacts with LEU64 and GLN154 were observed, suggesting that dynamic hydration also contributes to ligand stabilization (Figure 6D). Collectively, these results validate the docking predictions and confirm that asiatic acid binds stably to *IL-6* through a combination of hydrogen bonding and hydrophobic interactions, supporting its potential role as an *IL-6*-modulating anti-inflammatory agent.

**Figure 7:**
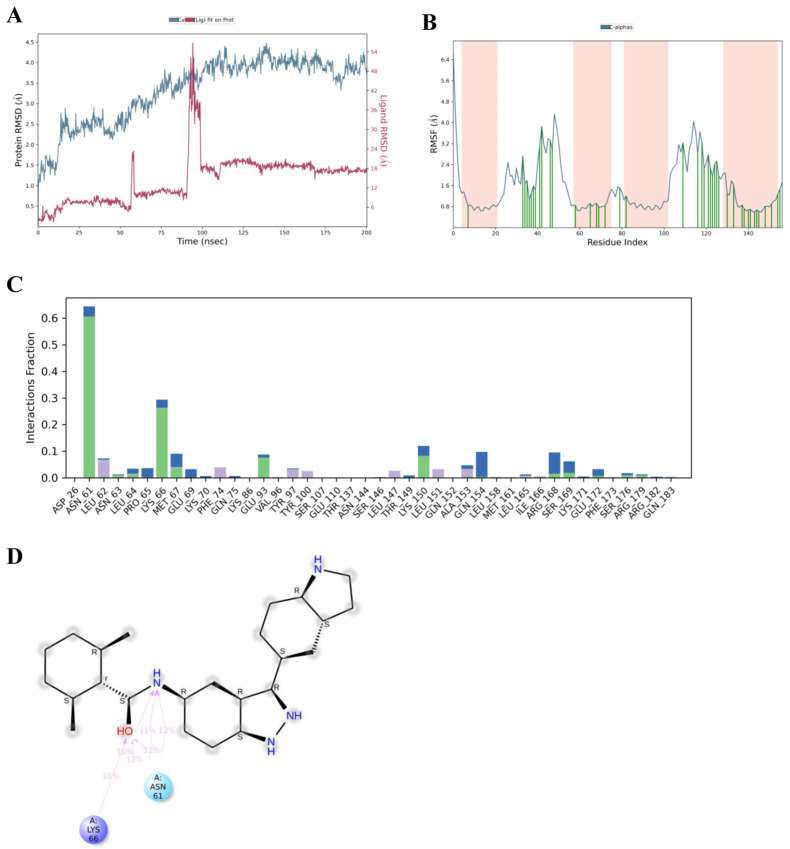
Molecular dynamics simulation of MD2-*TLR4*-IN-1-*IL-6* complex. (**A**) RMSD plot showing structural stability of *IL-6* (blue) and MD2-*TLR4*-IN-1 (red) over 200 ns simulation. (**B**) RMSF plot indicating fluctuation of Cα atoms across residues; pink shaded areas indicate binding site regions. (**C**) Interaction fraction plot showing frequency of ligand–residue contacts throughout simulation as Hydrogen Bonds (green), Hydrophobic (purple), and Water Bridges (blue). (**D**) Final 2D binding pose of MD2-*TLR4*-IN-1 with *IL-6* showing polar interaction with ASN61 (blue) and electrostatic interaction with the positively charged residue LYS66 (purple).

To provide a comparative assessment with asiatic acid, molecular dynamics simulations were also performed for MD2-*TLR4*-IN-1, a native *IL-6* inhibitor, in complex with *IL-6*. Over the 200 ns trajectory, the protein RMSD (Cα atoms) remained stable within 2.5–4.0 Å, indicating moderate conformational flexibility and overall structural stability of *IL-6*. The ligand RMSD (Lig fit Prot) initially stabilized at approximately 0.5–1.0 Å, suggesting strong and consistent binding. Although transient spikes were observed near 50 ns and 95–100 ns, these deviations were followed by rapid re-stabilization, indicating temporary reorientation within the binding pocket rather than unbinding (Figure 7A).

The RMSF profile for the MD2-*TLR4*-IN-1-*IL-6* complex revealed a pattern similar to that observed with asiatic acid, with most residues displaying low fluctuation amplitudes (<2 Å), suggesting good complex stability. Slightly elevated mobility was again noted in the loop regions around residues 35–50 and 115–130, consistent with flexible surface-exposed motifs involved in ligand accommodation (Figure 7B). Interaction fraction analysis revealed that ASN61 showed the highest interaction fraction among all residues, followed by LYS66, LYS150, GLN154, GLU93, ARG168, MET67, LEU62, SER169, ALA153, PHE74, PRO65, LEU64, GLU69, TYR97, TYR100, LEU147, LEU151, and GLU172, emphasizing their role as anchoring residues in *IL-6* binding (Figure 7C). The 2D protein–ligand contact map of MD2-*TLR4*-IN-1 further confirmed the presence of stable and recurrent hydrogen bonding with ASN61 and LYS66, two residues that were also critically involved in asiatic acid binding (Figure 7D). These interactions showed consistent occupancy over the trajectory, with multiple polar contacts anchoring the ligand. In contrast to asiatic acid, MD2-*TLR4*-IN-1 exhibited slightly fewer water-mediated interactions, suggesting a more rigid and direct engagement with key *IL-6* residues. Nonetheless, the shared contact residues between both ligands, especially ASN61 and LYS66, highlight the pharmacophoric significance of the AB-loop region in *IL-6* inhibition.

## 4. Discussion

Network pharmacology has emerged as a pivotal role in systems biology, providing a powerful framework for discovering novel drug targets and elucidating disease mechanisms at the level of interconnected biological targets and pathways, particularly in multifactorial disorders such as osteoporosis [26,27]. Accumulating experimental evidence indicates that asiatic acid influences bone remodeling by attenuating osteoclast activity and modulating inflammatory and metabolic signaling, particularly through *NF-κB*- and *PPARγ*-associated mechanisms, leading to reduced bone loss in experimental osteoporosis models [18,28,29]. However, osteoporosis pathogenesis is driven by coordinated interactions among multiple signaling pathways rather than by isolated molecular events. Consistent with this concept, GO and KEGG enrichment analyses in the present study highlighted the concurrent involvement of inflammatory response-related processes, *IL-6*/*STAT3* signaling, and PPAR signaling pathways, together with enrichment of Th17 cell differentiation and AGE–RAGE/PI3K–Akt-related pathways, suggesting functional crosstalk among inflammatory and metabolic networks that collectively regulate bone resorption and formation [7,9,10,30,31,32,33]. While these findings support the anti-osteoporotic potential of asiatic acid, they do not fully explain how these effects are coordinated at the network level or whether upstream regulatory nodes are involved. Network pharmacology therefore offers an appropriate strategy to examine the system-wide actions of asiatic acid and to identify key molecular hubs underlying its effects in osteoporosis.

Several key targets were identified through network pharmacology, including *IL-6*, *STAT3*, *PPARG*, *NFKB1*, *PTGS2*, *ESR1*, *TLR4*, *HSP90AB1*, *PPARA*, and *RELA*. These genes encode *IL-6*, *STAT3*, PPARy, *NF-κB* p105, COX-2, ESRα, *TLR4*, HSP90-β, PPARα, and *NF-κB* p65, respectively, which are critically involved in inflammation, hormone signaling, and bone remodeling. Among the identified targets, *IL-6*/*STAT3* signaling axis plays a central role in skeletal biology. *IL-6*, especially via trans-signaling with soluble *IL-6* receptor, activates gp130/JAK/*STAT3*, leading to increased RANKL in stromal/osteoblast cells and subsequent activation of nuclear factor of activated T-cells, cytoplasmic 1 (NFATc1) and osteoclast-specific genes in precursor cells. This axis is further amplified under estrogen-deficient conditions, linking it directly to postmenopausal bone loss [28,29,33]. In contrast, *STAT3* signaling in osteoblasts and osteocytes supports load-induced bone formation, highlighting the importance of cellular context in determining its net skeletal effect [34]. Clinically, the relevance of this pathway is underscored by evidence that *IL-6* blockade with tocilizumab lowers bone resorption and preserves bone mineral density in inflammatory conditions such as rheumatoid arthritis [35].

Additionally, *PPARγ* was identified as a key metabolic regulator associated with osteoporosis. Activation of *PPARγ* promotes adipogenic differentiation of bone marrow mesenchymal stem cells at the expense of osteoblastogenesis, increasing marrow fat and reducing bone formation [36]. Moreover, *PPARγ* facilitates osteoclastogenesis through PGC-1β-dependent activation of the NFATc1 pathway, boosting bone resorption. These molecular effects align with the clinical observations of increased fracture risk in patients treated with thiazolidinedione *PPARγ* agonists [37]. The *NF-κB* pathway also emerged as a critical signaling hub in osteoporosis. Activation of the p65/p50 heterodimer (where p50 is derived from the *NFKB1* precursor p105) downstream of RANKL-RANK signaling induces NFATc1 and osteoclast-specific genes, driving osteoclast differentiation [9,38]. Inflammatory cytokines and estrogen deficiency further enhance *NF-κB* activity, while its activation in osteoblast-lineage cells suppresses bone formation, collectively contributing to net bone loss [39,40]. Consistent with these mechanisms, previous experimental studies have demonstrated that asiatic acid protects against osteoporosis mainly by inhibiting osteoclastogenesis through suppression of *NF-κB* signaling. In RANKL-stimulated osteoclast precursors, asiatic acid reduced TRAP^+^ osteoclast formation, bone resorption, and nuclear translocation of *NF-κB* p65, thereby downregulating NFATc1 and osteoclast-specific genes; consistent protection against bone loss was observed in OVX mouse models [18,28]. Asiatic acid also upregulates Smad7, further antagonizing TGF-β and *NF-κB* signaling to attenuate OVX-induced bone resorption [28]. In addition to antiresorptive effects, asiatic acid suppresses *PPARγ* expression and adipogenic markers in bone marrow mesenchymal stromal cells, shifting differentiation toward osteoblastogenesis [17]. Collectively, these findings establish *NF-κB* p65 and *PPARγ* as experimentally validated hub targets of asiatic acid in osteoporosis, whereas direct modulation of *IL-6* and *STAT3* has remained insufficiently characterized. Given its central hub position in the network analysis, its convergence across enriched inflammatory pathways, and its well-established upstream role in inflammation-driven osteoclastogenesis, *IL-6* was prioritized for further molecular dynamics simulation to evaluate the stability and persistence of its interaction with asiatic acid under dynamic conditions.

Molecular docking analysis is widely employed in biological research and drug discovery to predict the binding affinity of ligands to receptor proteins [41]. In the present study, molecular docking analysis revealed that asiatic acid exhibited stronger binding affinities toward several osteoporosis-related proteins, including *STAT3*, *PPARγ*, *NF-κB* p105, COX-2, ESRα, *TLR4*, and *NF-κB* p65, compared to their native ligands. In contrast, for *IL-6*, the native ligand exhibited a slightly more negative predicted binding energy than asiatic acid, indicating a higher binding affinity. Nevertheless, asiatic acid was predicted to bind *IL-6* through hydrogen bonding with ASN61, GLU172, LEU62, and LYS66, along with hydrophobic interaction involving ARG168. Notably, GLU172, LYS66, and ARG168 have been reported as key residues targeted by MD2-*TLR4*-IN-1, a potential *IL-6* inhibitor that mitigates inflammation-driven bone loss in osteoporosis patients [42]. Furthermore, asiatic acid was predicted to interact with *STAT3* through hydrophobic interaction with PRO336, a residue similarly targeted by natural *STAT3* inhibitors [43]. Importantly, as *NF-κB* p65 plays a crucial in osteoclast differentiation [44], asiatic acid was predicted to bind directly to the DNA-binding subdomain of p65 through hydrogen bonds with ARG158 and HIS181, as well as a hydrophobic interaction with PRO182. Consistently, a curcuminoid derived from *Curcuma longa* has been shown to preferentially bind these same amino acid residues of *NF-κB* p65 in model of osteoclastogenesis [45]. These interactions suggest that binding of asiatic acid within the DNA-binding domain may interfere with the association of the p65-p50 heterodimer with nuclear DNA [45,46]. Nevertheless, molecular docking provides theoretical predictions of binding interactions, and experimental confirmation, such as biophysical assays, crystallographic studies, or site-directed mutagenesis, is needed to validate the precise interactions of asiatic acid with *IL-6*, *STAT3*, and *NF-κB* proteins.

Molecular dynamics simulation is a powerful computational technique widely used to evaluate the temporal stability, conformational flexibility, and interaction persistence of ligand–protein complexes in an explicit solvent environment [21,47]. To complement molecular docking results and provide a more realistic insight into the interaction between asiatic acid and *IL-6* under near-physiological conditions, a 200 ns MD simulation was conducted. Although molecular docking revealed that asiatic acid exhibited a strong binding affinity for *IL-6* (−8.64 kcal/mol), molecular dynamics simulation further demonstrated the stability and durability of this interaction over time. Notably, asiatic acid was predicted to bind with amino acid residues that overlap with those targeted by a known *IL-6* inhibitor [42], reinforcing its potential as an *IL-6* antagonist. Throughout the simulation, the asiatic acid-*IL-6* complex maintained a stable RMSD trajectory, remaining below 3 Å for most of the simulation period and exhibiting only minor fluctuations between 170–200 ns. These observations indicate that the ligand maintained a consistent and well-anchored binding conformation throughout the simulation period. The analysis also revealed that asiatic acid formed persistent interactions with ASN61, LEU62, and LYS66 located in the AB-loop, and ARG168 in helix D of the *IL-6* structure [48,49]. These structural motifs are known to constitute Site I, the primary binding interface between *IL-6* and its receptor IL-6Rα [49,50]. The AB-loop and helix D are essential for *IL-6* receptor recognition and downstream signaling [48,51]. Thus, asiatic acid’s engagement with these critical residues suggests that it may competitively interfere with *IL-6*–IL-6Rα complex formation, blocking receptor-mediated signal transduction and offering a plausible anti-inflammatory mechanism [52]. Consistent with this interpretation, similar residue hotspots have emerged in other *IL-6* inhibitor studies, as shown in molecular docking of dexamethasone, which showed binding to ASN60/ASN61, while thymoquinone, a natural anti-inflammatory compound, was shown to target ARG168, overlapping directly with the asiatic acid interaction site [52]. To further assess the relevance of asiatic acid’s interaction profile, MD simulation was also performed for MD2-*TLR4*-IN-1, a known *IL-6* inhibitor. Interestingly, both ligands interacted with common anchoring residues, ASN61, LEU62, LYS66, and ARG168, but asiatic acid displayed slightly greater flexibility while maintaining persistent binding. This suggests that asiatic acid may mimic the native ligand’s pharmacophore while offering additional adaptability within the binding pocket.

Collectively, these findings indicate a common pharmacophoric pattern among small-molecule *IL-6* inhibitors, wherein effective compounds anchor into the receptor-binding groove, stabilize local protein conformations, and reduce flexibility in the AB-loop and helix D regions through hydrogen bonding and hydrophobic interactions. This study, integrating network pharmacology, molecular docking, and molecular dynamics simulation, provides compelling mechanistic insight into asiatic acid’s potential as an *IL-6* modulator. The identification of *IL-6* as a potential upstream target of asiatic acid has important translational implications. Given that *IL-6* blockade reduces bone resorption in inflammatory diseases, asiatic acid may represent a natural, multi-target adjunct candidate for inflammation-driven osteoporosis, particularly in postmenopausal or chronic inflammatory settings [53]. Unlike conventional antiresorptive agents that primarily target osteoclast activity, asiatic acid appears to modulate upstream cytokine signaling, metabolic regulation, and transcriptional networks simultaneously, which may theoretically reduce compensatory pathway activation.

These findings provide a strong foundation for future drug development targeting inflammation-driven bone loss. In recent years, integrative in silico approaches have been increasingly recognized as valuable tools in natural product-based drug discovery, particularly for elucidating multi-target mechanisms and prioritizing candidates for experimental validation [54,55,56]. In this context, the present study employed an integrative in silico framework to investigate the potential anti-osteoporotic effects of asiatic acid. Nevertheless, such computational analyses are inherently hypothesis-generating in nature and require experimental validation to confirm their biological relevance. Therefore, the *IL-6*-specific anti-osteoporotic effects of asiatic acid remain to be validated in both in vitro and in vivo models. In vitro validation represents a critical next step, particularly using osteoblast and osteoclast cell models to directly assess the effects of asiatic acid on *IL-6*–mediated signaling pathways, including *STAT3* and *NF-κB*, as well as on functional endpoints related to bone resorption and formation. Despite the promising in silico results, it is important to acknowledge that favorable binding affinity does not necessarily translate to in vivo efficacy. Asiatic acid, as a pentacyclic triterpenoid, may exhibit limited oral bioavailability and tissue distribution [53]. Accordingly, optimization strategies, including formulation enhancement or structural modification, may be required to achieve therapeutically relevant concentrations in bone tissue. In vivo studies employing ovariectomized or inflammation-induced osteoporosis models will be essential to confirm efficacy and safety. Additionally, biophysical approaches such as surface plasmon resonance or isothermal titration calorimetry could directly verify *IL-6* binding, while transcriptomic or phosphoproteomic analyses may further clarify pathway-level effects [57,58].

## 5. Conclusions

This study highlights the potential anti-osteoporotic role of asiatic acid through an integrated in silico approach combining network pharmacology, molecular docking, and molecular dynamics simulation. Network pharmacology identified key osteoporosis-related targets involved in inflammatory signaling, hormone regulation, and bone remodeling, with key hubs including *IL-6*, *STAT3*, *NF-κB*, and *PPARγ*. Molecular docking revealed favorable binding affinities between asiatic acid and these targets, particularly *IL-6*. Molecular dynamics simulation further confirmed the structural stability of the asiatic acid-*IL-6* complex, demonstrating persistent protein–ligand interactions throughout the simulation period. Collectively, these findings suggest that asiatic acid may exert osteoprotective effects via multi-target regulation of inflammation-driven bone loss, extending its known downstream actions on *NF-κB* and *PPARγ* signaling to potential upstream regulation of *IL-6*-mediated pathways. By providing system-level and structural evidence supporting *IL-6* as a plausible molecular target of asiatic acid, this study advances the mechanistic understanding of its anti-osteoporotic potential. Although experimental validation is warranted, the present work establishes a robust mechanistic framework for future in vitro, in vivo, and translational studies and supports further exploration of asiatic acid as a natural multi-target therapeutic candidate for the prevention and management of osteoporosis.

## Figures and Tables

**Table 1:** The top 10 hub genes among the 135 overlapping targets of asiatic acid and osteoporosis.

Target	Common Name
72 kDa type IV collagenase	MMP2
Nitric oxide synthase, inducible	NOS2
C5a anaphylatoxin chemotactic receptor 1	C5AR1
G-protein coupled receptor 55	GPR55
Photoreceptor-specific nuclear receptor	NR2E3
Cyclin-dependent kinase 1	CDK1
Sex hormone-binding globulin	SHBG
Presequence protease, mitochondrial	PREP
Cholinesterase	BCHE
Prothrombin	THRB

**Table 2:** The Go and KEGG enrichment analysis of targets shared by asiatic acid and osteoporosis.

Description	Number of Genes	Pathway Genes	Fold Enrichment	Enrichment FDR
GO biological process				
GO:0006954 inflammatory response	52	892	9.9	2.84 × 10^−35^
GO:0071417 cellular response to organonitrogen compound	38	654	9.8	7.22 × 10^−25^
GO:0009725 response to hormone	47	898	8.9	1.87 × 10^−29^
GO:0010243 response to organonitrogen compound	54	1061	8.6	7.26 × 10^−34^
GO:0014070 response to organic cyclic compound	47	958	8.3	3.11 × 10^−28^
GO:1901701 cellular response to oxygen-containing compound	59	1272	7.9	2.83 × 10^−35^
GO:1901698 response to nitrogen compound	54	1172	7.8	9.74 × 10^−32^
GO:0033993 response to lipid	44	980	7.6	1.02 × 10^−24^
GO:1901700 response to oxygen-containing compound	75	1752	7.3	2.97 × 10^−44^
GO:0071495 cellular response to endogenous stimulus	58	1409	7.0	7.16 × 10^−32^
GO:0009719 response to endogenous stimulus	68	1660	6.9	2.11 × 10^−38^
GO:0042592 homeostatic proc.	59	1916	5.2	6.45 × 10^−26^
GO:0010033 response to organic substance	92	3269	4.8	5.49 × 10^−43^
GO:0070887 cellular response to chemical stimulus	92	3300	4.7	9.29 × 10^−43^
GO:0071310 cellular response to organic substance	72	2609	4.7	2.96 × 10^−30^
GO:0051239 reg. of multicellular organismal proc.	71	3024	4.0	2.08 × 10^−25^
GO:0009605 response to external stimulus	71	3073	3.9	5.33 × 10^−25^
GO:0065008 reg. of biological quality	94	4103	3.9	6.38 × 10^−37^
GO:0042221 response to chemical	106	4821	3.7	5.49 × 10^−43^
GO:0006950 response to stress	85	4424	3.3	3.24 × 10^−26^
GO cellular component				
GO:0008305 integrin complex	5	31	27.3	3.86 × 10^−5^
GO:0098636 protein complex involved in cell adhesion	5	53	16.0	2.94 × 10^−4^
GO:0043235 receptor complex	16	429	6.3	6.85 × 10^−7^
GO:0009897 external side of plasma membrane	13	459	4.8	1.10 × 10^−4^
GO:0005667 transcription regulator complex	15	553	4.6	3.86 × 10^−5^
GO:0043025 neuronal cell body	13	513	4.3	2.73 × 10^−4^
GO:0098552 side of membrane	19	757	4.3	8.23 × 10^−6^
GO:0005887 integral component of plasma membrane	37	1894	3.3	8.32 × 10^−9^
GO:0031226 intrinsic component of plasma membrane	38	1978	3.3	8.32 × 10^−9^
GO:0009986 cell surface	19	1050	3.1	3.00 × 10^−4^
GO:0042175 nuclear outer membrane–endoplasmic reticulum membrane network	23	1373	2.8	1.47 × 10^−4^
GO:0000785 chromatin	23	1392	2.8	1.71 × 10^−4^
GO:0005789 endoplasmic reticulum membrane	22	1351	2.8	3.00 × 10^−4^
GO:0098827 endoplasmic reticulum subcompartment	22	1355	2.8	3.00 × 10^−4^
GO:0005783 endoplasmic reticulum	36	2262	2.7	2.06 × 10^−6^
GO:0005694 chromosome	29	2003	2.5	1.40 × 10^−4^
GO:0031090 organelle membrane	49	4154	2.0	2.57 × 10^−5^
GO:0005654 nucleoplasm	52	4581	1.9	2.57 × 10^−5^
GO:0031982 vesicle	50	4466	1.9	5.43 × 10^−5^
GO:0031981 nuclear lumen	55	4973	1.9	2.57 × 10^−5^
GO molecular function				
GO:0004955 prostaglandin receptor activity	8	10	135.6	5.70 × 10^−15^
GO:0004954 prostanoid receptor activity	8	11	123.3	1.62 × 10^−14^
GO:0004953 icosanoid receptor activity	9	17	89.7	1.45 × 10^−14^
GO:0004879 nuclear receptor activity	18	57	53.5	2.23 × 10^−24^
GO:0098531 ligand-activated transcription factor activity	18	57	53.5	2.23 × 10^−24^
GO:0003707 nuclear steroid receptor activity	8	30	45.2	3.85 × 10^−10^
GO:0001223 transcription coactivator binding	9	42	36.3	1.71 × 10^−10^
GO:0005496 steroid binding	17	107	26.9	1.01 × 10^−17^
GO:0042562 hormone binding	10	93	18.2	8.62 × 10^−9^
GO:0001221 transcription coregulator binding	12	120	16.9	3.85 × 10^−10^
GO:0008289 lipid binding	32	841	6.4	2.45 × 10^−15^
GO:0033218 amide binding	17	481	6.0	1.44 × 10^−7^
GO:0008134 transcription factor binding	21	639	5.6	8.35 × 10^−9^
GO:0008270 zinc ion binding	26	947	4.7	2.62 × 10^−9^
GO:0038023 signaling receptor activity	52	1908	4.6	6.64 × 10^−20^
GO:0060089 molecular transducer activity	52	1908	4.6	6.64 × 10^−20^
GO:0046914 transition metal ion binding	33	1247	4.5	1.47 × 10^−11^
GO:0019899 enzyme binding	40	2237	3.0	4.12 × 10^−9^
GO:0046872 metal ion binding	59	4647	2.2	2.40 × 10^−8^
GO:0043169 cation binding	60	4737	2.1	1.77 × 10^−8^
KEGG				
Path:hsa05235 PD-L1 expression and PD-1 checkpoint pathway in cancer	11	89	20.9	2.99 × 10^−10^
Path:hsa03320 PPAR signaling pathway	9	75	20.3	1.12 × 10^−8^
Path:hsa05215 Prostate cancer	11	97	19.2	6.27 × 10^−10^
Path:hsa04931 Insulin resistance	11	108	17.3	1.47 × 10^−9^
Path:hsa05222 Small-cell lung cancer	9	92	16.6	5.13 × 10^−8^
Path:hsa04659 Th17 cell differentiation	10	108	15.7	1.50 × 10^−8^
Path:hsa04933 AGE-RAGE signaling pathway in diabetic complications	9	100	15.3	1.02 × 10^−7^
Path:hsa04919 Thyroid hormone signaling pathway	10	121	14.0	4.02 × 10^−8^
Path:hsa05418 Fluid shear stress and atherosclerosis	10	138	12.3	1.16 × 10^−7^
Path:hsa05163 Human cytomegalovirus infection	16	224	12.1	3.80 × 10^−11^
Path:hsa04613 Neutrophil extracellular trap formation	13	189	11.7	2.85 × 10^−9^
Path:hsa05206 MicroRNAs in cancer	11	161	11.6	4.48 × 10^−8^
Path:hsa05207 Chemical carcinogenesis-receptor activation	13	197	11.2	4.07 × 10^−9^
Path:hsa05205 Proteoglycans in cancer	13	202	10.9	5.05 × 10^−9^
Path:hsa05417 Lipid and atherosclerosis	13	214	10.3	9.45 × 10^−9^
Path:hsa05171 Coronavirus disease-COVID-19	14	232	10.2	2.85 × 10^−9^
Path:hsa05200 Pathways in cancer	31	530	9.9	7.08 × 10^−20^
Path:hsa04080 Neuroactive ligand–receptor interaction	19	362	8.9	3.80 × 10^−11^
Path:hsa04151 PI3K-Akt signaling pathway	17	354	8.1	1.42 × 10^−9^
Path:hsa05165 Human papillomavirus infection	15	331	7.7	1.93 × 10^−8^

**Table 3:** The binding affinities of asiatic acid and native ligands for the osteoporosis-related proteins.

Proteins	PDB ID	Asiatic Acid		Native Ligands	
		ΔG_docking_ (kcal/mol)	Ki (μM)	ΔG_docking_ (kcal/mol)	Ki (μM)
*IL-6*	1ALU	−8.64	0.46	−9.10	0.21
*STAT3*	6NJS	−6.17	30.19	−5.94	44.43
*PPARγ*	9CK0	−7.16	5.65	−5.78	57.93
*NF-κB* p105	8TQD	−7.70	2.28	−7.42	3.66
COX-2	5F1A	−6.86	9.35	−7.57	2.85
ESRα	4XI3	−5.98	41.60	−5.60	79.15
*TLR4*	3FXI	−5.87	50.2	−5.11	179.47
HSP90-β	5UC4	−5.57	83.30	−5.81	55.00
PPARα	1K7L	−6.41	20.13	−6.70	12.33
*NF-κB* p65	1NFI	−6.15	30.95	−5.70	65.81

**Table 4:** The binding interactions between amino acid residues of osteoporosis-related proteins and asiatic acid with the interaction distances.

Protein	Interaction	Residue	Distance (Å)	Same Amino Acid Residue with Native Ligand
*IL-6*	Hydrogen bond	ASN61, GLU172, LEU62, LYS66	1.86, 1.71/2.32, 1.87, 3.48	GLU172, LYS66
	Hydrophobic	ARG168	3.79	ARG168
*STAT3*	Hydrogen bond	THR346, PRO330	2.81, 1.96	-
	Hydrophobic	PRO336	1.96	-
*PPARγ*	Hydrogen bond	LYS474, TYR477, GLN451, ASP475	1.73, 3.07, 2.81/2.54, 1.98	-
*NF-κB* p105	Hydrogen bond	LYS51, LYS79, GLY68, SER74, TYR81	1.82, 3.03/2.92, 1.75, 2.07, 3.60	-
COX-2	Hydrogen bond	LYS79, PRO84	2.12, 3.77	-
ESRα	Hydrogen bond	GLN500, LEU495	2.41, 2.03/2.25	-
*TLR4*	Hydrogen bond	SER534, LYS560, LYS561, GLN510	2.48, 2.39, 2.20, 1.88	-
	Hydrophobic	LEU511	3.73	-
Hsp90-β	Hydrogen bond	GLN23, PHE32	2.05, 2.42	GLN23
PPARα	Hydrogen bond	LYS208, GLN461, HIS274	2.09, 2.10, 3.22	-
	Hydrophobic	HIS274	3.89	-
*NF-κB* p65	Hydrogen bond	ARG158, LEU179, GLN247, HIS181	3.06/1.71, 2.08/2.09, 1.93, 3.48	-
	Hydrophobic	PRO182	4.00	-

“-” denotes the absence of shared interacting amino acid residues with the native ligand.

## Data Availability

The original contributions presented in this study are included in the article/Supplementary Material. Further inquiries can be directed to the corresponding author.

## Supplementary Materials

The following supporting information can be downloaded at: https://www.mdpi.com/article/10.3390/medsci14010041/s1, Figure S1: 2D molecular docking analysis illustrating the interactions between native ligands (blue) and their osteoporosis protein targets; Figure S2: 3D molecular docking analysis illustrating the interactions between native ligands (blue) and its osteoporosis protein targets; Table S1: The 135 overlapping targets of asiatic acid and osteoporosis; Table S2: The binding interactions between amino acid residue for inflammation-related proteins and their native ligands with the interaction distances.

## Abbreviations

The following abbreviations are used in this manuscript:

Å	Angström
COX-2	Cyclooxygenase-2
ΔG	Binding free energy
ESRα	Estrogen receptor alpha
FDR	False discovery rate
GAFF	General AMBER force field
GO	Gene Ontology
*HSP90AB1*	Heat shock protein 90 beta
*IL-6*	Interleukin-6
IL-6Rα	Interleukin-6 receptor alpha
KEGG	Kyoto Encyclopedia of Genes and Genomes
Ki	Inhibitory constant
MD	Molecular dynamics
*NF-κB*	Nuclear factor kappa B
PPARα	Peroxisome proliferator-activated receptor alpha
*PPARγ*	Peroxisome proliferator-activated receptor gamma
PPI	Protein–protein interaction
RANKL	Receptor activator of nuclear factor kappa B ligand
RMSD	Root mean square deviation
RMSF	Root mean square fluctuation
SEA	Similarity Ensemble Approach
*STAT3*	Signal transducer and activator of transcription 3
STRING	Search Tool for the Retrieval of Interacting Genes/Proteins
*TLR4*	Toll-like receptor 4
