# Supplementary material for: In Silico Investigation Reveals IL-6 as a Key Target of Asiatic Acid in Osteoporosis: Insights from Network Pharmacology, Molecular Docking, and Molecular Dynamics Simulation
Source: Med Sci (Basel). 2026 Jan 15;14(1):41. doi: 10.3390/medsci14010041 (PMC12821701; doi:10.3390/medsci14010041)
Supplement: Supplementary file 1 [file medsci-14-00041-s001.zip › medsci-4084982-supplementary.pdf]

Supplementary data

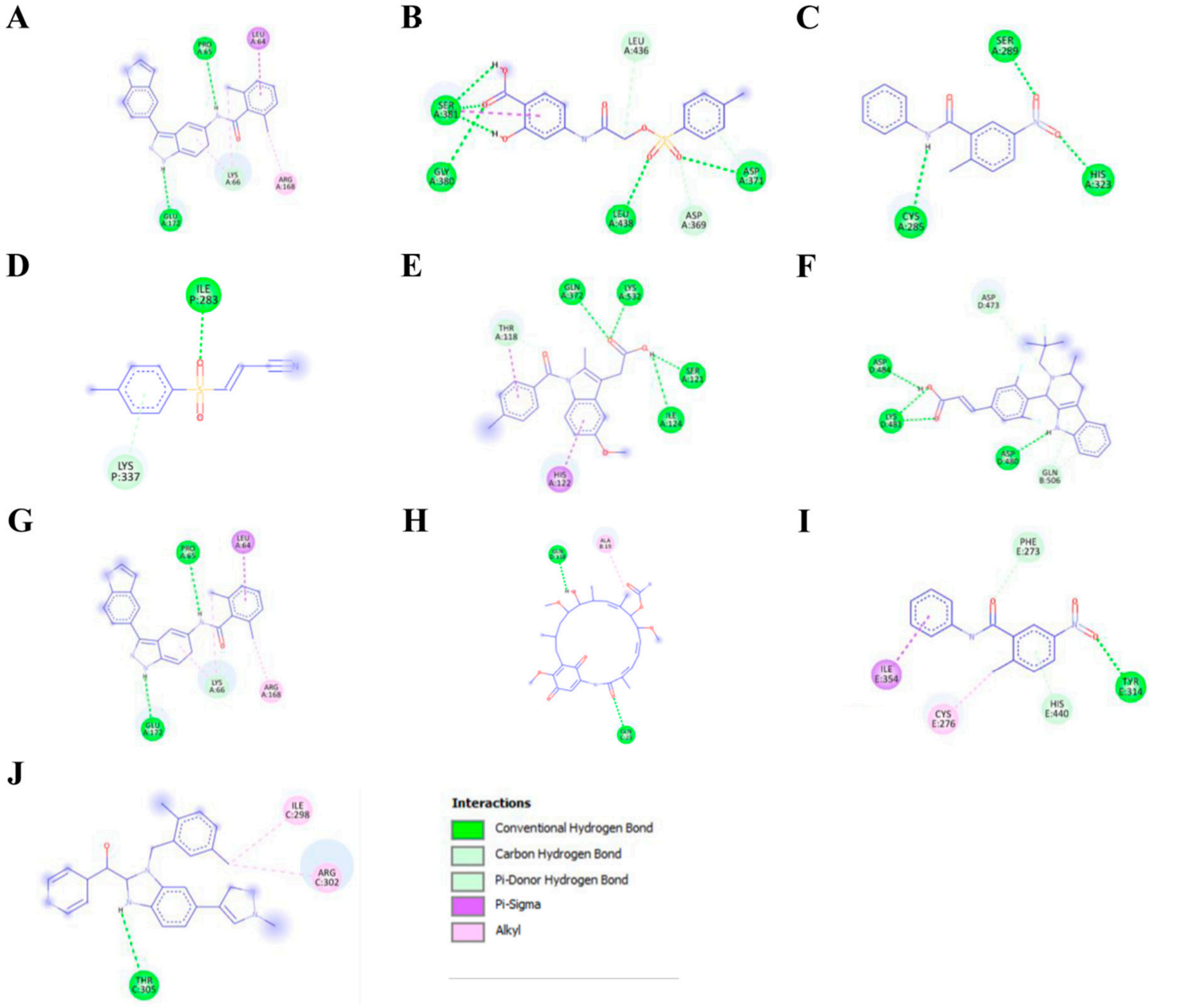

Figure S1 2D molecular docking analysis illustrating the interactions between native ligands (blue) and its osteoporosis protein targets. Panels illustrate interaction maps between (A) MD2-TLR4-IN-1 and IL-6, (B) NSC 74859 and STAT3, (C) mifobate and PPARγ, (D) BAY 11-7082 and NF-KB p50, (E) Indomethacin and COX-2, (F) AZD9496 and ESR1, (G) MD2-IN-1 and TLR4, (H) geldanamycin and HSP90AB1, (I) GW9662 and PPARa, and (J) UCB-9260 and NF-KB p65.

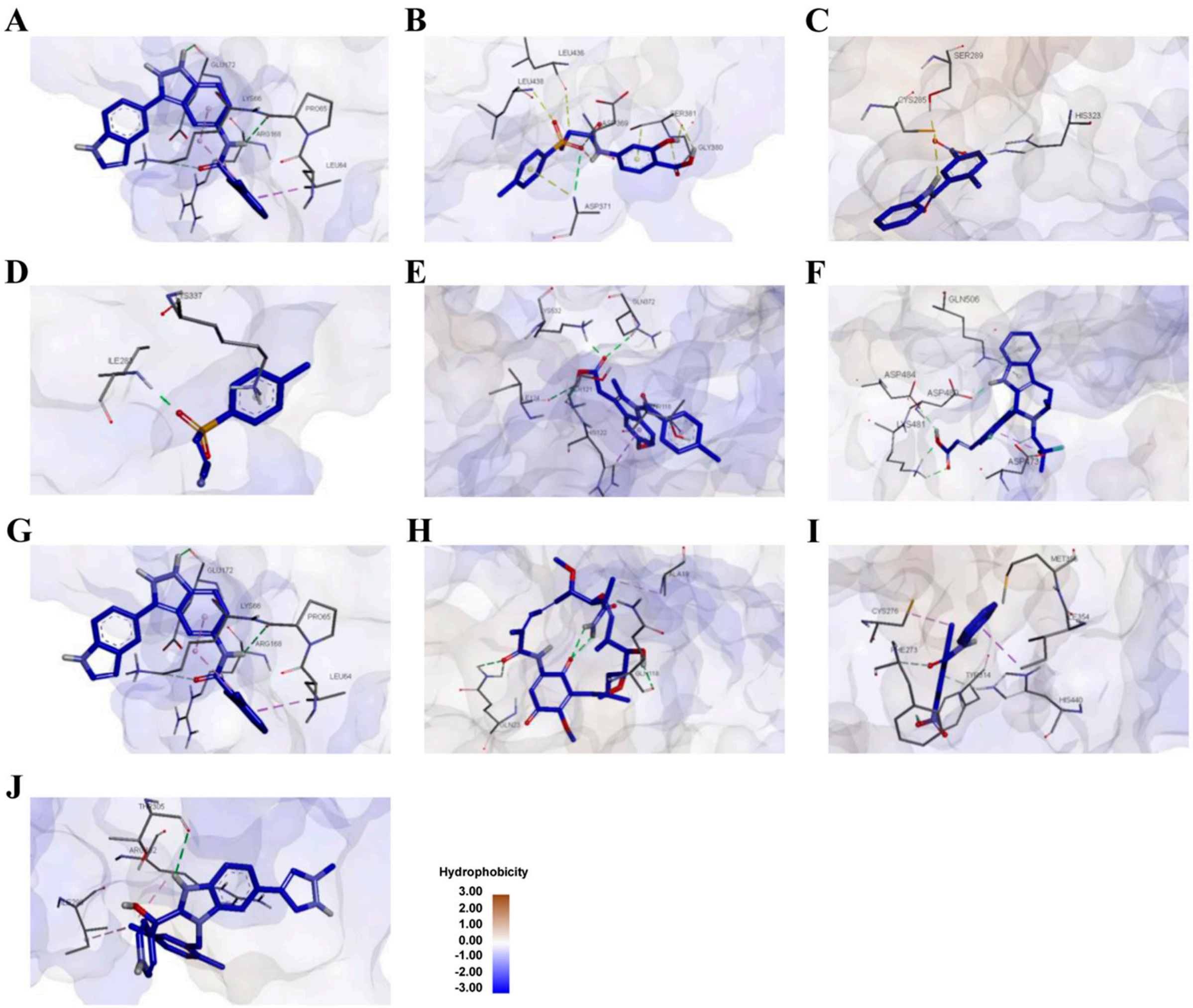

Figure S2 3D molecular docking analysis illustrating the interactions between native ligands (blue) and its osteoporosis protein targets. Panels illustrate interaction maps between (A) MD2-TLR4-IN-1 and IL-6, (B) NSC 74859 and STAT3, (C) mifobate and PPARγ, (D) BAY 11-7082 and NF-KB p50, (E) Indomethacin and COX-2, (F) AZD9496 and ESR1, (G) MD2-IN-1 and TLR4, (H) geldanamycin and HSP90AB1, (I) GW9662 and PPARa, and (J) UCB-9260 and NF-KB p65. Pink, green, and purple dashed lines represent hydrophobic interactions, hydrogen bonds, and π–σ interactions, respectively.

Table S1. The 135 overlapping targets of asiatic acid and osteoporosis

Target	Common name
72 kDa type IV collagenase	MMP2
Nitric oxide synthase, inducible	NOS2
C5a anaphylatoxin chemotactic receptor 1	C5AR1
G-protein coupled receptor 55	GPR55
Photoreceptor-specific nuclear receptor	NR2E3
Cyclin-dependent kinase 1	CDK1
Sex hormone-binding globulin	SHBG
Presequence protease, mitochondrial	PREP
Cholinesterase	BCHE
Prothrombin	THRB
Receptor-type tyrosine-protein phosphatase N2	PTPN2
Prostaglandin E2 receptor EP1 subtype	PTGER1
Nuclear factor erythroid 2-related factor 2	NFE2L2
Aromatase	CYP19A1
Glucagon receptor	GCGR
Lysine-specific demethylase 6B	KDM6B
Thymidylate synthase	TYMS
Neprilysin	MME
Interstitial collagenase	MMP1
fMet-Leu-Phe receptor	FPR1
Peroxisome proliferator-activated receptor delta	PPARD
Stromelysin-1	MMP3
Hsc70-interacting protein	F13A1
Hepatic sodium/bile acid cotransporter	SLC10A1
Prostaglandin E2 receptor EP2 subtype	PTGER2
Arachidonate 5-lipoxygenase-activating protein	ALOX5AP

Target	Common name
E3 ubiquitin-protein ligase Mdm2	MDM2
Nuclear receptor subfamily 4 immunity group A member 1	NR4A1
Glucose-6-phosphate 1-dehydrogenase	G6PD
Krueppel-like factor 5	KLF5
cGMP-specific 3',5'-cyclic phosphodiesterase	PDE5A
Coagulation factor X	F10
Tyrosine-protein phosphatase non-receptor type 6	PTPN6
Progesterone receptor	PGR
Thyroid hormone receptor alpha	THRA
Excitatory amino acid transporter 1	SLC1A3
Low molecular weight phosphotyrosine protein phosphatase	ACP1
Phosphatidylinositol 3-kinase regulatory subunit alpha	PIK3R1
Platelet-derived growth factor receptor alpha	PDGFRA
N-formyl peptide receptor 2	FPR2
Prostaglandin E2 receptor EP3 subtype	PTGER3
Prostaglandin D2 receptor	PTGDR
Prostaglandin D2 receptor	PLCG1
Ferroportin	SLC40A1
Mitogen-activated protein kinase kinase kinase 14	MAP3K14
Adenosylhomocysteinase	AHCY
Prostaglandin F2-alpha receptor	PTGFR
Fatty-acid amide hydrolase 1	FAAH
Lysine-specific histone demethylase 1A	KDM1A
Fatty acid-binding protein, heart	FABP3
Cytosolic phospholipase A2	PLA2G4A
Toll-like receptor 8	TLR8
Stearoyl-CoA desaturase	SCD

Target	Common name
Signal transducer and activator of transcription 3	STAT3
Sphingosine kinase 1	SPHK1
Induced myeloid leukemia cell differentiation protein Mcl-1	MCL1
Interleukin-6	IL6
DNA polymerase beta	POLB
Tyrosine-protein phosphatase non-receptor type 1	PTPN1
Cannabinoid receptor 2	CNR2
Integrin alpha-IIb	ITGA2B
DNA topoisomerase 2-alpha	TOP2A
Transcription factor p65	RELA
Cystic fibrosis transmembrane conductance regulator	CFTR
Toll-like receptor 4	TLR4
Toll-like receptor 9	TLR9
Fatty acid-binding protein, adipocyte	FABP4
11-beta-hydroxysteroid dehydrogenase 1	HSD11B1
Tissue factor	F3
Glycogen phosphorylase, muscle form	PYGM
Androgen receptor	AR
NPC intracellular cholesterol transporter 1	NPC1
Cytochrome P450 3A4	CYP3A4
11-beta-hydroxysteroid dehydrogenase type 2	HSD11B2
Chromatin remodeling regulator CECR2	CES2
Ubiquitin carboxyl-terminal hydrolase 7	USP7
Prostaglandin E synthase	PTGES
3-oxo-5-alpha-steroid 4-dehydrogenase 2	SRD5A2
Voltage-dependent T-type calcium channel subunit alpha-1H	CACNA1H
Integrin beta-1	ITGB1
Prostaglandin G/H synthase 2	PTGS2

Target	Common name
Estrogen receptor	ESR1
3',5'-cyclic-AMP phosphodiesterase 4D	PDE4D
cGMP-inhibited 3',5'-cyclic phosphodiesterase 3A	PDE3A
DNA repair nuclease/redox regulator APEX1	APEX1
Prostacyclin receptor	PTGIR
Prostaglandin E2 receptor EP4 subtype	PTGER4
3-hydroxy-3-methylglutaryl-coenzyme A reductase	HMGCR
Mineralocorticoid receptor	NR3C2
Melanocortin receptor 4	MC4R
G-protein coupled bile acid receptor 1	GPBAR1
Adenosine receptor A3	ADORA3
Heat shock protein HSP 90-beta	HSP90AB1
Telomerase reverse transcriptase	TERT
Steroid 17-alpha-hydroxylase/17,20 lyase	CYP17A1
cAMP-dependent protein kinase catalytic subunit alpha	PRKACA
Nuclear receptor ROR-gamma	RORC
Nuclear factor NF-kappa-B p105 subunit	NFKB1
Lysosomal protective protein	CTSA
P2X purinoceptor 7	P2RX7
Estrogen receptor beta	ESR2
Sodium-dependent serotonin transporter	SLC6A4
Corticosteroid-binding globulin	SERPINA6
Polyunsaturated fatty acid 5-lipoxygenase	ALOX5
Oxysterols receptor LXR-alpha	NR1H3
Tyrosine-protein phosphatase non-receptor type 11	PTPN11
Peroxisome proliferator-activated receptor gamma	PPARG
Glucocorticoid receptor	NR3C1
Serine/threonine-protein kinase mTOR	MTOR

Target	Common name
C-C chemokine receptor type 2	CCR2
RecQ-like DNA helicase BLM	BLM
Integrin alpha-V	ITGAV
Inhibitor of nuclear factor kappa-B kinase subunit alpha	CHUK
Disintegrin and metalloproteinase domain-containing protein 10	ADAM10
Type-1 angiotensin II receptor	AGTR1
Cathepsin D	CTSD
Ileal sodium/bile acid cotransporter	SLC10A2
Leukotriene B4 receptor 1	LTB4R
Integrin alpha-4	ITGA4
Peroxisome proliferator-activated receptor alpha	PPARA
Nuclear receptor ROR-alpha	RORA
Beta-secretase 1	BACE1
Fatty acid-binding protein, liver	FABP1
Adenosine receptor A1	ADORA1
Glutamine synthetase	GLUL
Histone deacetylase 2	HDAC2
Voltage-dependent calcium channel subunit alpha-2/delta-2	CA2
CD81 antigen	CD81
Aminopeptidase N	ANPEP
Bile acid receptor	NR1H4
Cyclin-dependent kinase 5	CDK5
Integrin beta-3	ITGB3
Proteasome subunit beta type-1	PSMB1
Prostaglandin G/H synthase 1	PTGS1
Proteasome subunit beta type-9	PSMB9

Table S2. The binding interactions between amino acid residue for inflammation-related proteins and their native ligands with the interaction distances

**Native ligand**	**Protein target**	**Interaction**	**Residue**	**Distance (Å)**
MD2-TLR4-IN-1	IL-6	Hydrogen bond	PRO65, GLU172, LYS66,	2.76, 2.76, 3.12,
		Hydrophobic	LEU64, LYS66, ARG168	3.69, 3.58/3.50, 3.68
NSC 74859	STAT3	Hydrogen bond	GLY380, SER381, LEU438, ASP369, LEU436, ASP371	3.22, 3.31/2.13/2.30, 2.68, 2.69, 3.61, 3.56
		Hydrophobic	SER381	3.69
Mifobate	PPARy	Hydrogen bond	SER289, HIS323, CYS285	1.94, 2.01, 2.72
BAY 11-7082	NF-κB p150	Hydrogen bond	ILE283, LYS337	1.91, 3.00
Indomethacin	COX-2	Hydrogen bond	GLN372, LYS532, SER121, ILE124, THR118	2.51, 1.96, 2.00, 3.06, 3.29
		Hydrophobic	THR118, HIS122	3.39, 3.43
AZD9496	ESR*α*	Hydrogen bond	ASP480, ASP484, ASP473, GLN506	1.87, 2.20, 3.77, 2.87/3.35
MD2-IN-1	TLR4	Hydrogen bond	SER140, GLU142, PHE144	3.69, 2.89, 3.14
Geldanamycin	HSP90-β	Hydrogen bond	GLN23, GLN118	2.58/2.74, 2.18
		Hydrophobic	ALA19	3.58
GW9662	PPARα	Hydrogen bond	SER289, HIS323, CYS285	1.94, 2.01, 2.72
UCB-9260	NF-κB p65.	Hydrogen bond	THR305	2.89
		Hydrophobic	ILE298, ARG302	3.65, 3.92

Å angstrom
